# Circadian rhythm of COPD symptoms in clinically based phenotypes. Results from the STORICO Italian observational study

**DOI:** 10.1186/s12890-019-0935-2

**Published:** 2019-09-09

**Authors:** Scichilone Nicola, Antonelli Incalzi Raffaele, Blasi Francesco, Schino Pietro, Cuttitta Giuseppina, Zullo Alessandro, Ori Alessandra, Canonica Giorgio Walter, on behalf of STORICO study group, Pietro Schino, Giuseppina Cuttitta, Maria Pia Foschino, Renato Prediletto, Carmelindo Mario Enrico Tranfa, Maria Cristina Zappa, Pasquale Patriciello, Luciana Labate, Salvatore Mariotta, Stefano Nava, Alessandro Vatrella, Michele Mastroberardino, Riccardo Sarzani, Antonio Iuliano, Lamberto Maggi, Anna Zedda, Alberto Pesci, Giuseppe Sera, Antonello Nicolini, Di Donato Salvatore Walter, Silvia Forte, Del Donno Mario, Federica Rivolta, Mauro Ferliga, Antonio Filippo Raco, Di Re Luigi, Gaetano Cabibbo, Rosario Maselli, Carlo Gulotta, Stefano Nardini, Enrico Eugenio Guffanti, Walter Castellani, Luca Triolo, Giovanni Passalacqua, Bianca Beghè, Lo Cicero Salvatore, Enzo Faccini, Elena Atzeni, Roberto Tazza, Piercarlo Giamesio

**Affiliations:** 1 DIBIMIS, University of Palermo, Piazza delle Cliniche, 2, 90127 Palermo, Italy; 2 University Biomedical Campus of Rome, via Alvaro del Portillo, 21, 00128 Rome, Italy; 3 Internal Medicine Department, Respiratory Unit and Cystic Fibrosis Adult Center Fondazione IRCCS Cà Granda Ospedale Maggiore Policlinico and Department of Pathophysiology and Transplantation, University of Milan, via Francesco Sforza, 35, 20122 Milan, Italy; 4 Miulli Hospital, Acquaviva delle FontiStrada Prov. 127 Acquaviva – Santeramo Km. 4, 10070021 Bari, Italy; 5 National Research Council, via Ugo La Malfa, 153, 90146 Palermo, Italy; 6 Medineos Observational Research, Viale Virgilio 54/U, 41123 Modena, Italy; 7 Personalized Medicine Asthma and Allergy Clinic Humanitas University Humanitas research Hospital Rozzano (Milan), via Manzoni, 56, 20089 Rozzano, MI Italy

**Keywords:** 24-hour symptoms, Clinical phenotype, Respiratory function, Real-world

## Abstract

**Background:**

Chronic Obstructive Pulmonary Disease (COPD) encompasses various phenotypes that severely limit the applicability of precision respiratory medicine. The present investigation is aimed to assess the circadian rhythm of symptoms in pre-defined clinical COPD phenotypes and its association with health-related quality of life (HR-QoL), the quality of sleep and the level of depression/anxiety in each clinical phenotype.

**Methods:**

The STORICO (NCT03105999) Italian observational prospective cohort study enrolled COPD subjects. A clinical diagnosis of either chronic bronchitis (CB), emphysema (EM) or mixed COPD-asthma (MCA) phenotype was made by clinicians at enrollment. Baseline early-morning, day-time and nocturnal symptoms (gathered via the *Night-time, Morning and Day-time Symptoms of COPD questionnaire*), HR-QoL (via the *St. George’s Respiratory Questionnaire*), anxiety and depression levels (via the *Hospital Anxiety and Depression Scale*), quality of sleep (via *COPD and Asthma Sleep Impact Scale*), physical activity (via the *International Physical Activity Questionnaire*) as well as lung function were recorded.

**Results:**

606 COPD subjects (age 71.4 ± 8.2 years, male 75.1%) were studied. 57.9, 35.5 5.3 and 1.3% of the sample belonged to the CB, EM, MCA and EM + CB phenotypes respectively. The vast majority of subjects reported early-morning and day-time symptoms (79.5 and 79.2% in the CB and 75.8 and 77.7% in the EM groups); the proportion suffering from night-time symptoms was higher in the CB than in the EM group (53.6% vs. 39.5%, *p* = 0.0016). In both CB and EM, indiscriminately, the presence of symptoms during the 24-h day was associated with poorer HR-QoL, worse quality of sleep and higher levels of anxiety/depression.

**Conclusions:**

The findings highlight the primary classificatory role of nocturnal symptoms in COPD.

**Trial registration:**

Trial registration number: NCT03105999, date of registration: 10th April 2017.

## Background

Chronic Obstructive Pulmonary Disease (COPD) is a common, preventable condition characterized by persistent airflow limitation, initiated and sustained by chronic exposure to smoke and other irritants [1]. The complexity and the heterogeneous clinical presentation of the disease, however, requires a more comprehensive approach, obliging physicians to take account of symptoms and exacerbations, and to manage the concomitant occurrence of extra-pulmonary pathological conditions. It is evident, on this basis, that Burrows and Fletcher’s original proposal of emphysematous and bronchitic phenotypes [2] is no longer sufficient to identify the different clinical forms of the disease in clinical practice. A COPD phenotype is considered to be a set of attributes that can help differentiate patients on the basis of clinically meaningful parameters, such as symptoms, exacerbations, progression of disease decline, physical inactivity, response to treatment, and mortality.

One effort to classify COPD individuals according to their clinical presentations is the Spanish COPD guidelines [3], which distinguish the emphysema from the chronic bronchitic phenotype and also consider the rate of exacerbations. In addition, a mixed COPD-asthma phenotype (asthma-COPD overlap [ACO]) has been proposed, although its interpretation and association with exacerbation and mortality are now controversial [4–6]. Distinguishing the bronchitic from the emphysematous phenotype is apparently an easy task. The former is marked by productive cough and early onset of hypoxemia, with high prevalence of respiratory failure and chronic *cor pulmonale*, whereas the latter displays early and severe dyspnea with late-onset hypoxemia. Hypothetically, these clinical presentations could benefit selectively by the treatment options available, but, in real life settings the spectrum of therapies is variably distributed across the range of severity of COPD, without any distinction in terms of phenotypes, thus making such personalized care treatment more difficult.

Taken together, these observations show the need for deeper understanding of the clinical phenotypes of COPD. The STORICO study (STudio Osservazionale sulla caratteRizzazione dei sIntomi delle 24 ore nei pazienti con broncopneumopatia cronica ostruttiva, *Observational study on characterization of 24-h symptoms in patients with COPD*) offers a unique opportunity to analyze the clinical characteristics of subjects with COPD in relation to the 24-h day-long variability of symptoms. For example, given that dyspnea depends mainly on physical effort, it is logical to expect this symptom to be far more evident during daytime, and in EM subjects.

To reinforce the classical phenotyping strategy, this paper aims primarily to assess, within the clinical COPD phenotypes, the circadian rhythm of COPD symptoms at baseline (in terms of frequency of early-morning, day-time and night-time symptoms). As secondary objective, for each clinical phenotype, we investigated the potential association at enrollment of the circadian variability of symptoms with both disease severity and selected indicators of health status.

## Method

### Study design

STORICO (NCT03105999) is an Italian, multicenter observational prospective cohort study conducted in 40 pulmonology referral care centers. The enrollment started in February 2016 and the 1-year longitudinal phase of the study ended in June 2018; a total of three visits (baseline and follow-up at 6-month intervals) were performed. The present paper reports the results of the cross-sectional phase. More details about the STORICO study are available elsewhere [7].

### Subjects

Subjects of both sexes aged ≥50, current or former smokers with a smoking history of at least 10 pack-years, with a diagnosis of COPD and in stable conditions for at least 12 months according to GOLD 2014 [8] were enrolled consecutively. Patients provided written, informed consent before participation. Patients participating in a clinical trial and those who had changed their COPD treatment regimen in the 3 months prior to enrolment were excluded, as were patients with recent exacerbations (within 1 month prior to enrollment). Also, patients under continuous use of oxygen therapy or suffering from asthma, sleep apnea syndrome or other chronic diseases that reduced life expectancy to less than 3 years (Charlson index> 3) were excluded. Among enrolled patients, only those with available information on the frequency of COPD symptoms during each part of the 24-h day were analyzed.

The sample size was determined by criteria of feasibility. In fact, given the volume of patients handled by the centers involved in the study, inclusion of approximately 600 subjects with the characteristics defined by the Inclusion/Exclusion criteria was deemed reasonable. An evaluation of the possible precision of the estimates was performed in consideration of the primary objectives, finding that a sample of 100 patients per group (i.e. per class of phenotype) would allow precise estimates of the expected proportion of COPD symptoms ≥30%. More details concerning sample size justification are provided elsewhere [7].

### Clinical assessment

On the first assessment day, anthropometric data and clinical history were collected and each subject was assigned a clinical COPD phenotype based on the judgment of the clinician: *chronic bronchitis* (CB), defined as the presence of productive cough for at least 3 months in two consecutive years; *emphysema* (EM), for patients whose predominant symptoms are dyspnea and reduced tolerance to exercise; *mixed COPD-asthma* (MCA) for patients with documented not completely reversible airflow obstruction, accompanied by symptoms or signs of obstruction reversibility [9, 10]. As part of the investigation, local researchers were asked to express their degree of confidence in the choice of clinical phenotype on a scale of 0 to 10, from no confidence at all to maximum of confidence. Disease severity was defined using the combined COPD assessment according to GOLD [11]. Spirometry was performed according to the recommendations of the American Thoracic Society (ATS) and the European Respiratory Society (ERS); forced expiratory volume in the first second (FEV_1_) and forced vital capacity (FVC) were retained for analysis. Although not mandatory by protocol, the centers were invited to record static lung volumes, and Residual Volume (RV) and Total Lung Capacity (TLC) were obtained together with diffusion capacity for carbon monoxide (DLCO), in order to support the clinical assignment of subjects to the phenotypes.

### Multidimensional assessment

According to the study design, subjects completed the *Night-time, Morning and Day-time Symptoms of COPD questionnaire* [12]). This is a 33-item questionnaire developed by Almirall S.A., Barcelona, Spain covering the frequency and severity of COPD symptoms (breathlessness, coughing, bringing up phlegm or mucus, chest tightness, chest congestion and wheezing) during each part of the day. This represented the primary outcome of the study.

The questionnaire includes 13 symptom items for night-time (i.e. the time between going to bed and getting up), 10 symptom items for early-morning (i.e. the time from getting up until approximately 11:00 am) and 10 symptom items for day-time (i.e. from approximately 11:00 am until the subject goes to bed).

To address the secondary outcomes of the study the following measures were performed: the level of perceived breathlessness and the extent to which it affected mobility were assessed by the *modified Medical Research Council* (mMRC) dyspnea scale ranging from 0 (breathless with strenuous exercise) to 4 (too breathless to leave the house or breathless when dressing or undressing) [13]; health-related quality of life (HR-QoL) was evaluated by the *St. George’s Respiratory Questionnaire* (SGRQ), including the subject’s perception of recent respiratory problems (Symptoms component), disturbances to daily physical activity (Activity component) and disturbances of psycho-social function (Impact component); a total score was also calculated. Scores range from 0 (no impairment) to 100 (highest impairment) [14–17], anxiety and depression states were investigated through the *Hospital Anxiety and Depression Scale* (HADS) [18–20], with a total score (emotional distress) ranging from 0 to 42; anxiety and depression subscale scores (range 0–21) were also computed, higher scores indicating more distress; the impact of respiratory symptoms on sleep was assessed by the *COPD and Asthma Sleep Impact Scale* (CASIS) [21], a self-administered, 7-item scale. The total score range is 0–100, with higher scores indicating greater sleep impairment in the previous week; physical activity was assessed with the *International Physical Activity Questionnaire* (IPAQ) [22, 23] and a categorical score (low, medium, high level of physical activity) was calculated.

The presence of relevant comorbidities and the number of exacerbations/year in the 5 years before enrollment were recorded. In particular, the presence of anemia was defined as hemoglobin < 13.5 g/dl (for males) and < 12 g/dl (for females), and an estimated glomerular filtration rate (eGFR) < 60 mL/min/1.73 m^2^ was considered as indicator of chronic kidney disease [24]. The eGFR was calculated according to the equation proposed by the Chronic Kidney Disease Epidemiology Collaboration [25].

### Statistical analysis

Descriptive statistics were calculated both overall and within each phenotype: mean and standard deviation (SD), median, interquartile range (IQR) for quantitative variables and absolute and relative frequency for categorical variables.

Missing values were not replaced and did not contribute to the analysis of the variable. Frequencies of missing data were given for all analyzed variables.

#### Primary objective

The frequency of COPD symptoms at enrolment was calculated overall and within each class of phenotype as the ratio between the number of patients with at least 1 (early-morning, day-time and night-time) symptom in the week before enrollment and the total number of evaluable patients in the class. Among the symptomatic patients, the proportion suffering from mild, moderate, severe and very severe symptoms in the week before enrollment was also calculated (overall and by clinical phenotype). The 95% confidence intervals for the proportions were provided when relevant.

#### Secondary objective

The association between the circadian variability of COPD symptoms and disease severity at enrollment was evaluated by calculating the frequency of patients with early-morning, day-time and night-time symptoms in the groups of patients according to disease severity (groups A to D of Global Initiative for Chronic Obstructive lung Disease (GOLD)2014 combined assessment). HR-QoL (SGRQ scores), quality of sleep (CASIS score), anxiety and depression (HADS scores), level of physical activity (IPAQ categorical score) at enrollment in patients with vs without early-morning, day-time and night-time symptoms were described and compared using T-test and Chi-square or Fisher exact test (in the case of non-parametric distribution). The significance threshold was adjusted for multiple comparisons (Bonferroni correction applied) and set to 0.0005 (0.05/number of tests). Site monitoring, data management and statistical analysis were performed by MediNeos an IQVIA Company (Modena, Italy). Statistical analysis was performed using SAS v9.4 and Enterprise Guide v7.1. More details about the STORICO methodology are available elsewhere [7].

## Results

### Subject characteristics

**Fig. 1:**
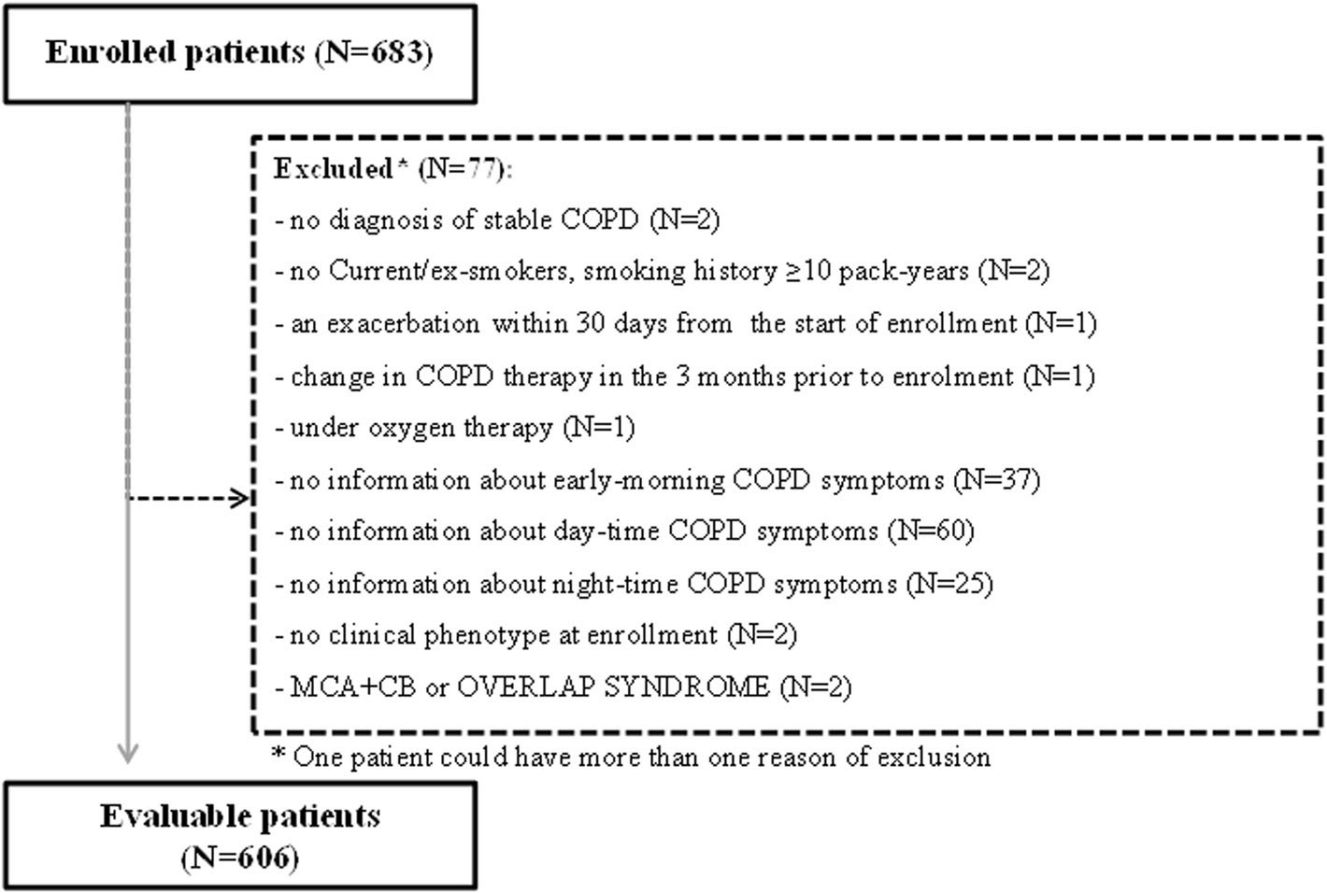
Patients enrolled and included in the analyses. CB: Chronic Bronchitis; MCA: Mixed-COPD asthma

Among the 683 COPD individuals enrolled in the STORICO study, 606 (88.7%) subjects (age 71.4 ± 8.2 years, 75.1% males) were deemed eligible for the analysis at enrollment; violations causing exclusion are shown in Fig. 1*.* A total of 351 subjects (57.9%) were classified as CB, 215 (35.5%) as EM and 32 (5.3%) as MCA. In addition, 8 subjects (1.3%) were classified as EM + CB and not retained for analysis due to the paucity of data. When asked for their level of confidence in attributing the phenotype, the vast majority of physicians (77.0% CB, 78.4% EM, 58.6% MCA) rated it at least ≥6 out of 10. Baseline socio-demographic and clinical characteristics (including ongoing therapies for COPD) for each clinical phenotype are shown in Table 1. Table 2 describes the clinical and functional characteristics of subjects according to phenotype. By comparison with EM phenotype, CB patients were more commonly overweight and had more exacerbations/year in the 5 years before baseline (median (IQR): 2 [1–4] in CB vs. 1 (0–2) in EM). As shown in Table 3, the SGRQ components and the total score were similar across phenotypes and, similarly, the CASIS score and the HADS scores did not differ between groups. On the other hand, low levels of physical activity were observed for 35.4% of CB and 18.1% of EM subjects.

**Table 1:** Socio-demographic, clinical and biological characteristics of the subjects

	Overall (*N* = 606)	CB (*N* = 351)	EM (*N* = 215)	MCA (*N* = 32)
Age (yrs) (mean ± SD)	71.4 ± 8.2	71.6 ± 8.3	71.5 ± 7.9	70.1 ± 8.2
Males (N, %)	455 (75.1)	256 (72.9)	174 (80.9)	20 (62.5)
Education (N, %)				
None	4 (0.7)	3 (0.9)	0	0
Primary school	173 (29.6)	111 (32.8)	54 (25.7)	7 (22.6)
Middle school	241 (41.2)	127 (37.6)	93 (44.3)	19 (61.3)
High school	133 (22.7)	76 (22.5)	51 (24.3)	4 (12.9)
University	34 (5.8)	21 (6.2)	12 (5.7)	1 (3.2)
*NK*	*21*	*13*	*5*	*1*
Occupational status (N, %)				
Unemployed	23 (3.8)	16 (4.6)	5 (2.4)	2 (6.5)
Employed	77 (12.8)	44 (12.6)	25 (11.7)	5 (16.1)
Retired	468 (77.9)	265 (75.9)	176 (82.6)	22 (71.0)
Housewife/househusband	33 (5.5)	24 (6.9)	7 (3.3)	2 (6.5)
*NK*	*5*	*2*	*2*	*1*
Smoking status (N, %)				
Former smoker	445 (73.4)	250 (71.2)	166 (77.2)	25 (78.1)
Current smoker	161 (26.6)	101 (28.8)	49 (22.8)	7 (21.9)
Estimated amount of tobacco consumed (pack/year) (median IQR)	39.0 (20.0–50.0)	35.0 (20.0–52.0)	40.0 (23.0–50.0)	30.0 (20.0–45.0)
Smoking duration (yrs) (median IQR)	40.0 (30.0–49.5)	40.0 (30.0–50.0)	40.0 (32.0–50.0)	39.0 (30.0–42.0)
BMI (N, %)				
Underweight (BMI < 18.5)	13 (2.2)	4 (1.2)	9 (4.2)	0
Normal weight (BMI 18.5–24.9)	183 (30.5)	90 (26.0)	81 (37.9)	11 (34.4)
Overweight (BMI 25–29.9)	272 (45.3)	163 (47.1)	91 (42.5)	16 (50.0)
Obese (BMI ≥30)	132 (22.0)	89 (25.7)	33 (15.4)	5 (15.6)
*NK*	*6*	*5*	*1*	*0*
Comorbidities (N, %)				
≥ 1 comorbidity	446 (73.6)	254 (72.4)	163 (75.8)	23 (71.9)
Anemia^a^	36 (26.7)	26 (33.8)	8 (16.0)	2 (33.3)
Arterial Hypertension	308 (50.8)	188 (53.6)	97 (45.1)	17 (53.1)
Atrial fibrillation	34 (5.6)	20 (5.7)	12 (5.6)	2 (6.3)
Cardiac ischemic disease	63 (10.4)	35 (10.0)	21 (9.8)	6 (18.8)
Diabetes	65 (10.7)	40 (11.4)	20 (9.3)	4 (12.5)
GERD	24 (4.0)	14 (4.0)	8 (3.7)	2 (6.3)
Neoplastic disease	31 (5.1)	10 (2.8)	17 (7.9)	3 (9.4)
Osteoporosis	26 (4.3)	19 (5.4)	5 (2.3)	1 (3.1)
Blood tests				
eGFR < 60 mL/min/1.73 m^2^ (N, %)	*n* = 122	*n* = 65	*n* = 49	*n* = 6
	17 (13.9)	11 (16.9)	4 (8.2)	2 (33.3)
Hemoglobin (g/dl) (mean ± SD)	*n* = 135	*n* = 77	*n* = 50	*n* = 6
	14.0 ± 1.7	13.8 ± 1.8	14.4 ± 1.4	13.2 ± 1.1
Mean cell volume (fl) (mean ± SD)	*n* = 106	*n* = 64	*n* = 35	*n* = 6
	90.8 ± 7.9	90.4 ± 7.7	91.2 ± 7.7	93.0 ± 11.4
Eosinophil count (n/mm^3^) (median IQR)	*n* = 129	*n* = 75	*n* = 47	*n* = 6
	2.3 (0.1–166.0)	3.0 (0.1–190.0)	4.0 (0.1–166.0)	0.1 (0.0–0.1)
Serum creatinine (mg/dL) (median IQR)	*n* = 122	*n* = 65	*n* = 49	*n* = 6
	0.9 (0.7–1.1)	0.9 (0.8–1.1)	0.8 (0.7–1.0)	0.8 (0.7–1.2)
Ongoing therapies for COPD (N, %)				
≥ 1	525 (86.6)	311 (88.6)	183 (85.1)	27 (84.4)
Triple therapy (LABA, LAMA, ICS)	187 (30.9)	116 (33.0)	62 (28.8)	7 (21.9)
LABA+LAMA	143 (23.6)	58 (16.5)	75 (34.9)	8 (25.0)
LAMA Alone	90 (14.9)	66 (18.8)	23 (10.7)	1 (3.1)
ICS + LABA	78 (12.9)	53 (15.1)	17 (7.9)	8 (25.0)
LABA Alone	18 (3.0)	12 (3.4)	4 (1.9)	2 (6.3)
Other	9 (1.5)	6 (1.7)	2 (0.9)	1 (3.1)

Data about EM + CB not shown

*BMI* body mass index. *CB* Chronic Bronchitis. *eGFR* estimated glomerular filtration rate. *EM* Emphysema. *GERD* Gastroesophageal Reflux Disease. *ICS* Inhaled corticosteroids. *IQR* interquartile range. *LABA* long-acting beta-agonist. *LAMA* long-acting muscarinic antagonists. *MCA* Mixed-COPD asthma. *NK* Unknown. *SD* standard deviation; Triple therapy includes any combination of LABA, LAMA, ICS

^a^Presence of anemia was evaluated for 135 patients in the total sample, 77 CB, 50 EM, 6 MCA

Percentages computed out of non-missing responses

**Table 2:** COPD medical history, lung function parameters and mMRC Scale score at enrollment

	Overall (*N* = 606)	CB (*N* = 351)	EM (*N* = 215)	MCA (*N* = 32)
COPD duration (yrs) (mean ± SD)	7.8 ± 6.5	7.7 ± 6.5	8.0 ± 6.3	8.7 ± 8.9
Age at COPD diagnosis (yrs) (mean ± SD)	63.6 ± 9.1	63.9 ± 9.4	63.5 ± 8.1	61.4 ± 11.5
N of COPD exacerbations/year (5 years before baseline) median (IQR)	2.0 (1.0–3.0)	2.0 (1.0–4.0)	1.0 (0.0–2.0)	3.0 (1.0–5.0)
COPD assessment (GOLD guidelines) (N, %)				
group A	155 (25.6)	76 (21.7)	66 (30.7)	12 (37.5)
group B	187 (30.9)	133 (37.9)	43 (20.0)	7 (21.9)
group C	126 (20.8)	59 (16.8)	57 (26.5)	8 (25.0)
group D	138 (22.8)	83 (23.6)	49 (22.8)	5 (15.6)
FEV1 (L) Median (IQR)	*n* = 535	*n* = 298	*n* = 200	*n* = 29
	1.5 (1.2–2.0)	1.6 (1.2–2.0)	1.5 (1.1–2.0)	1.5 (1.2–1.9)
FEV1 of the predicted (%) Median (IQR)	*n* = 537	*n* = 300	*n* = 200	*n* = 29
	63.9 (50.0–80.0)	66.9 (51.0–81.8)	61.9 (48.1–78.0)	58.4 (48.3–77.2)
FVC (L) Median (IQR)	*n* = 536	*n* = 299	*n* = 200	*n* = 29
	2.7 (2.2–3.4)	2.6 (2.2–3.3)	2.9 (2.2–3.5)	2.5 (2.1–3.1)
FEV1/FVC (%) Median (IQR)	*n* = 537	*n* = 300	*n* = 200	*n* = 29
	59.7 (49.0–69.0)	62.0 (52.0–70.0)	56.0 (45.0–66.6)	62.3 (53.0–68.1)
RV (L) Median (IQR)	*n* = 291	*n* = 130	*n* = 138	*n* = 21
	3.2 (2.6–4.2)	2.9 (2.3–3.7)	3.5 (2.9–4.8)	3.4 (2.8–4.9)
IC (L) Median (IQR)	*n* = 306	*n* = 143	*n* = 138	*n* = 24
	2.6 (2.1–3.4)	2.6 (2.1–3.3)	2.7 (2.1–3.5)	2.4 (2.2–3.3)
TLC (L) Median (IQR)	*n* = 286	*n* = 128	*n* = 135	*n* = 21
	6.4 (5.4–7.3)	6.0 (4.9–6.9)	6.8 (5.8–7.6)	6.5 (5.7–7.3)
RV/TLC Median (IQR)	*n* = 289	*n* = 128	*n* = 138	*n* = 21
	0.5 (0.4–0.6)	0.5 (0.4–0.6)	0.6 (0.5–0.6)	0.6 (0.5–0.6)
DLCO of the predicted (%) Median (IQR)	*n* = 205	*n* = 98	*n* = 92	*n* = 15
	68.0 (51.0–83.0)	69.5 (59.0–87.0)	63.0 (46.8–76.5)	76.7 (48.0–81.5)
mMRC Scale score	*n* = 577	*n* = 334	*n* = 205	*n* = 30
Median (IQR)	1.0 (1.0–2.0)	1.0 (1.0–2.0)	1.0 (1.0–2.0)	1.0 (1.0–2.0)
≥ 2 (N, %)	262 (45.4)	150 (44.9)	97 (47.3)	11 (36.7)

Data about EM + CB not shown

*CB* Chronic Bronchitis. *DLCO* diffusing capacity of the lung for carbon monoxide. *EM* Emphysema

*FEV1* forced expiratory volume in the first second. *FVC* forced vital capacity. *IC* Inspiratory capacity. *IQR* interquartile range. *MCA* Mixed-COPD asthma. *mMRC* modified Medical Research Council. *RV* Residual Volume. *SD* standard deviation. *TLC* Total lung capacity

**Table 3:** Quality of life, quality of sleep, level of physical activity and anxiety/depression at enrollment

	Overall	CB	EM	MCA
*N*	*582*	*339*	*204*	*32*
SGRQ symptoms score	42.5 ± 22.2	43.3 ± 22.7	40.7 ± 21.1	44.4 ± 25.3
activity score	49.8 ± 21.3	50.4 ± 21.4	49.1 ± 21.2	48.5 ± 22.1
impacts score	23.5 ± 18.7	24.0 ± 18.7	22.7 ± 18.8	22.5 ± 18.2
total score	34.5 ± 18.1	35.1 ± 18.2	33.5 ± 18.1	33.9 ± 18.5
*N*	*597*	*344*	*213*	*32*
CASIS total score	17.4 ± 16.2	19.3 ± 16.6	14.5 ± 14.9	14.3 ± 17.1
*N*	*566*	*332*	*196*	*30*
HADS Total score	9.5 ± 6.8	9.9 ± 6.9	8.9 ± 6.4	8.8 ± 7.1
Anxiety score	4.9 ± 3.7	5.1 ± 3.9	4.4 ± 3.3	4.8 ± 3.9
Depression score	4.7 ± 3.7	4.8 ± 3.6	4.5 ± 3.8	4.0 ± 3.6
*N*	*199*	*113*	*72*	*11*
IPAQ Level of physical activity (N, %)				
Low	57 (28.6)	40 (35.4)	13 (18.1)	3 (27.3)
Moderate	139 (69.8)	72 (63.7)	57 (79.2)	8 (72.7)
High	3 (1.5)	1 (0.9)	2 (2.8)	0 (0.0)

Data about EM + CB not shown

Mean ± SD (standard deviation) were shown

*CASIS* COPD and Asthma Sleep Impact Scale. *CB* Chronic Bronchitis. *EM* Emphysema. *HADS* Hospital Anxiety and Depression Scale. *IPAQ* International Physical Activity Questionnaire. *MCA* Mixed-COPD asthma. *SGRQ* St. George’s Respiratory Questionnaire

### Circadian rhythm of symptoms in the clinical COPD phenotypes

A higher proportion of patients suffered from respiratory symptoms in the early-morning and day-time (79.5 and 79.2% in CB and 75.8 and 77.7% in EM groups reported early-morning and day-time symptoms, respectively) than during night-time..

**Fig. 2:**
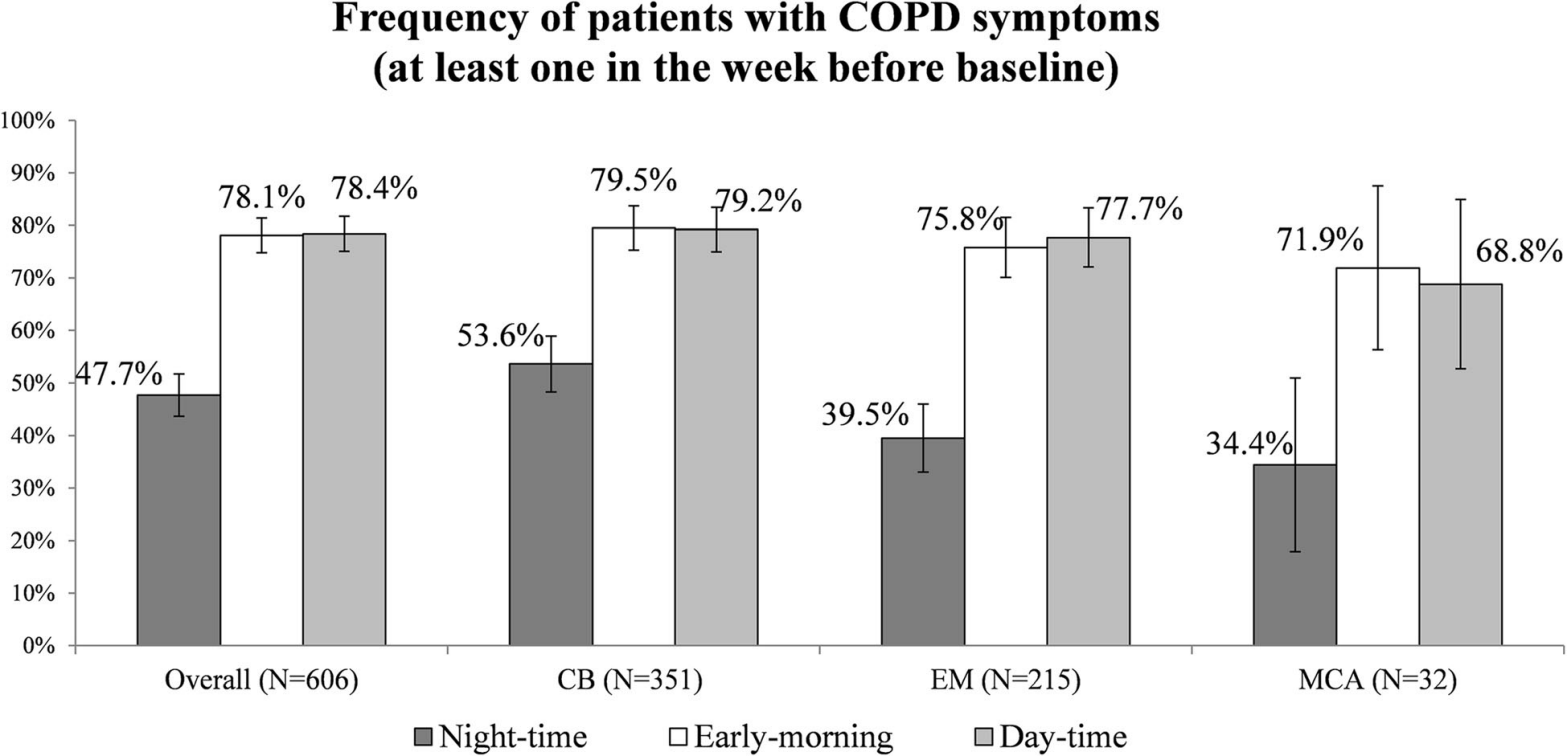
Frequency of patients with COPD symptoms (in the week before baseline). CB: Chronic Bronchitis; EM: Emphysema; MCA: Mixed-COPD asthma. 95% confidence intervals are shown

Interestingly, the proportion of subjects suffering from night-time symptoms was higher in the CB group (53.6%) than in the EM (39.5%) or MCA (34.4%) groups (Chi-square test *p*-value presence/absence of night-time symptoms vs clinical phenotype = 0.0016 – see Fig. 2). In the subsample of patients reporting night-time symptoms, the frequency of current smokers was not significantly higher in CB (*n* = 53, 28.2%) than in EM (*n* = 22, 25.9%) patients (Chi-square test smoke vs clinical phenotype p-value > 0.05). In addition, the prevalence of current smokers was unrelated to nocturnal symptoms in either CB (*n* = 53, 28.2% and *n* = 48, 29.4%, symptomatic vs asymptomatic patients), EM (*n* = 22, 25.9% and *n* = 27, 20.8%), or CB/EM (*n* = 75, 27.5% and *n* = 75, 25.6%).

**Fig. 3:**
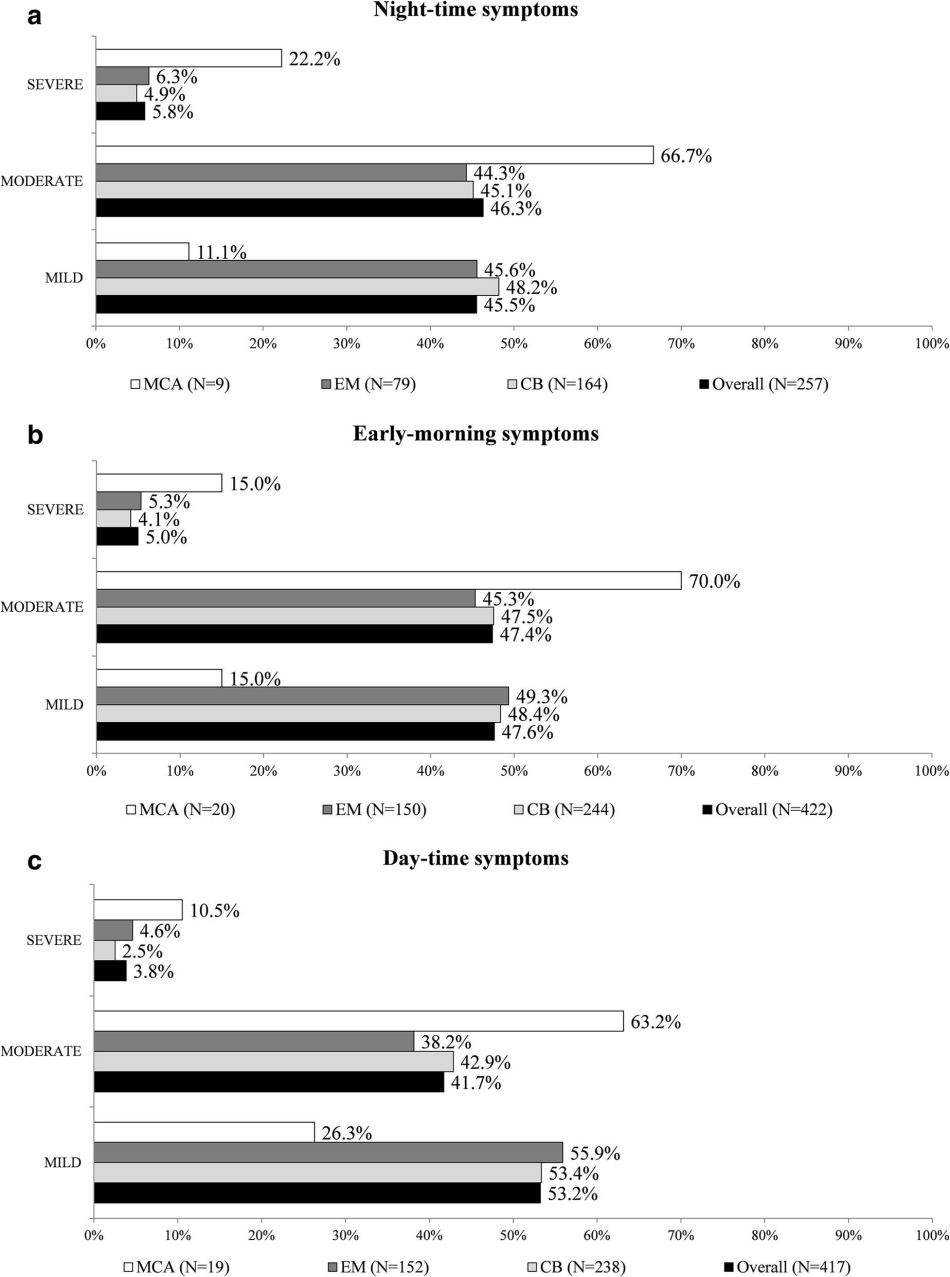
**a** Frequency of patients according to severity of night-time COPD symptoms (in the week before baseline). CB: Chronic Bronchitis; EM: Emphysema; MCA: Mixed-COPD asthma. Percentages computed out of patients with symptoms and with non-missing severity. **b** Frequency of patients according to severity of early-morning COPD symptoms (in the week before baseline). CB: Chronic Bronchitis; EM: Emphysema; MCA: Mixed-COPD asthma. Percentages computed out of patients with symptoms and with non-missing severity. **c** Frequency of patients according to severity of day-time COPD symptoms (in the week before baseline). CB: Chronic Bronchitis; EM: Emphysema; MCA: Mixed-COPD asthma. Percentages computed out of patients with symptoms and with non-missing severity

As shown in Fig. 3a, b and c, the CB and EM phenotypes did not differ in terms of severity of respiratory symptoms for any portion of the 24-h day. The higher proportion of MCA patients with severe and moderate symptoms in each portion of the day may depend on the small sample size.

### Association between circadian variability of symptoms in each clinical phenotype and severity of disease, HR-QoL, quality of sleep, level of physical activity and depression/anxiety

In each clinical phenotype, the frequency of symptoms increased significantly with the severity of COPD (in CB GOLD A-D vs. presence/absence of symptoms *p*-values < 0.0005 – see Table 4). In EM, the proportions of patients of groups A to D with early-morning, day-time and night-time symptoms were similar to those observed in CB, although the association between GOLD classification and symptoms was not statistically significant.

**Table 4:** Symptoms and severity of disease, HR-QoL, quality of sleep, level of physical activity and depression/anxiety

	EARLY-MORNING SYMPTOMS			DAY-TIME SYMPTOMS			NIGHT-TIME SYMPTOMS		
	No	Yes	p-value	No	Yes	p-value	No	Yes	p-value
A. CB (*N* = 351)									
GOLD combined COPD assessment (N, %)									
*n*	*72*	*279*		*73*	*278*		*163*	*188*	
GROUP A	28 (36.8)	48 (63.2)	**<.0001**	25 (32.9)	51 (67.1)	**0.0002**	52 (68.4)	24 (31.6)	**<.0001**
GROUP B	29 (21.8)	104 (78.2)		34 (25.6)	99 (74.4)		65 (48.9)	68 (51.1)	
GROUP C	6 (10.2)	53 (89.8)		8 (13.6)	51 (86.4)		24 (40.7)	35 (59.3)	
GROUP D	9 (10.8)	74 (89.2)		6 (7.2)	77 (92.8)		22 (26.5)	61 (73.5)	
SGRQ scores (mean ± SD)									
*n*	*71*	*268*		*71*	*268*		*159*	*180*	
symptoms	19.4 ± 16.2	49.7 ± 19.7	**<.0001**	18.5 ± 14.7	49.9 ± 19.6	**<.0001**	31.4 ± 20.3	53.9 ± 19.2	**<.0001**
activity	40.4 ± 17.6	53.0 ± 21.6	**<.0001**	39.0 ± 17.6	53.4 ± 21.4	**<.0001**	42.2 ± 19.0	57.6 ± 20.9	**<.0001**
impacts	14.6 ± 12.0	26.5 ± 19.4	**<.0001**	13.4 ± 12.0	26.8 ± 19.2	**<.0001**	15.7 ± 13.1	31.3 ± 19.9	**<.0001**
total	23.4 ± 12.6	38.2 ± 18.2	**<.0001**	22.1 ± 12.4	38.5 ± 17.9	**<.0001**	26.3 ± 13.8	42.9 ± 18.0	**<.0001**
CASIS scores (mean ± SD)									
*n*	*70*	*274*		*70*	*274*		*161*	*183*	
total	10.7 ± 12.3	21.5 ± 16.8	<.0001	9.1 ± 11.1	21.9 ± 16.7	< 0.0001	9.3 ± 9.7	28.1 ± 16.4	<.0001
HADS scores (mean ± SD)									
*n*	*69*	*263*		*69*	*263*		*156*	*176*	
total	8.3 ± 6.2	10.4 ± 7.0	0.0279	7.1 ± 5.9	10.7 ± 7.0	**< 0.0001**	8.0 ± 6.2	11.7 ± 7.1	**< 0.0001**
*n*	*70*	*266*		*70*	*266*		*157*	*179*	
anxiety	4.4 ± 3.5	5.3 ± 4.0	> 0.05	3.8 ± 3.4	5.5 ± 4.0	0.0018	4.1 ± 3.5	6.0 ± 4.0	**< 0.0001**
*n*	*69*	*265*		*69*	*265*		*156*	*178*	
depression	3.8 ± 3.0	5.1 ± 3.7	0.009	3.3 ± 3.0	5.2 ± 3.6	**< 0.0001**	3.9 ± 3.1	5.6 ± 3.8	**< 0.0001**
IPAQ Level of physical activity (N, %)									
*n*	*17*	*96*		*19*	*94*		*50*	*63*	
Low	4 (10.0)	36 (90.0)	> 0.05	4 (10.0)	36 (90.0)	> 0.05	19 (47.5)	21 (52.5)	> 0.05
Moderate	12 (16.7)	60 (83.3)		14 (19.4)	58 (80.6)		30 (41.7)	42 (58.3)	
High	1 (100.0)	0 (0)		1 (100.0)	0 (0)		1 (100.0)	0 (0)	
B. EM (*N* = 215)									
GOLD combined COPD assessment (N, %)									
*n*	*52*	*163*		*48*	*167*		*130*	*85*	
GROUP A	24 (36.4)	42 (63.6)	0.0009	23 (34.8)	43 (65.2)	0.0075	48 (72.7)	18 (27.3)	0.0035
GROUP B	14 (32.6)	29 (67.4)		10 (23.3)	33 (76.7)		22 (51.2)	21 (48.8)	
GROUP C	11 (19.3)	46 (80.7)		11 (19.3)	46 (80.7)		39 (68.4)	18 (31.6)	
GROUP D	3 (6.1)	46 (93.9)		4 (8.2)	45 (91.8)		21 (42.9)	28 (57.1)	
SGRQ scores (mean ± SD)									
*n*	*50*	*154*		*46*	*158*		*122*	*82*	
symptoms	20.8 ± 14.7	47.1 ± 18.6	**<.0001**	23.7 ± 17.8	45.6 ± 19.3	**<.0001**	33.2 ± 18.9	51.7 ± 19.2	**<.0001**
activity	39.7 ± 20.8	52.2 ± 20.5	**0.0003**	34.8 ± 17.4	53.3 ± 20.4	**<.0001**	43.7 ± 18.7	57.2 ± 22.3	**<.0001**
impacts	13.9 ± 11.5	25.5 ± 19.9	**<.0001**	11.1 ± 8.7	26.0 ± 19.7	**<.0001**	16.1 ± 12.7	32.5 ± 22.0	**<.0001**
total	22.7 ± 13.0	37.0 ± 18.1	**<.0001**	20.3 ± 10.6	37.4 ± 18.0	**<.0001**	27.1 ± 13.3	43.0 ± 20.0	**<.0001**
CASIS scores (mean ± SD)									
*n*	*51*	*162*		*47*	*166*		*129*	*84*	
total	7.2 ± 8.2	16.8 ± 15.8	**<.0001**	7.1 ± 10.0	16.6 ± 15.4	**< 0.0001**	8.0 ± 8.7	24.6 ± 16.8	**<.0001**
HADS scores (mean ± SD)									
*n*	*45*	*151*		*42*	*154*		*121*	*75*	
total	6.5 ± 5.5	9.6 ± 6.5	0.0039	5.1 ± 5.1	9.9 ± 6.3	**< 0.0001**	7.2 ± 5.6	11.7 ± 6.6	**< 0.0001**
*n*	*46*	*153*		*44*	*155*		*123*	*76*	
anxiety	3.4 ± 3.0	4.8 ± 3.3	0.0079	2.7 ± 2.4	4.9 ± 3.3	**< 0.0001**	3.8 ± 3.0	5.5 ± 3.5	**0.0002**
*n*	*48*	*152*		*43*	*157*		*123*	*77*	
depression	3.3 ± 3.3	4.9 ± 3.9	0.0107	2.6 ± 3.2	5.0 ± 3.8	**0.0002**	3.4 ± 3.3	6.2 ± 3.9	**< 0.0001**
IPAQ Level of physical activity (N, %)									
*n*	*17*	*55*		*15*	*57*		*54*	*18*	
Low	2 (15.4)	11 (84.6)	> 0.05	1 (7.7)	12 (92.3)	0.0265	8 (61.5)	5 (38.5)	> 0.05
Moderate	14 (24.6)	43 (75.4)		12 (21.1)	45 (78.9)		44 (77.2)	13 (22.8)	
High	1 (50.0)	1 (50.0)		2 (100.0)	0 (0)		2 (100.0)	0 (0)	

The percentages of the GOLD combined COPD assessment are computed by row, i.e. over the total number of patients in Groups A, B, C, D respectively. Similarly, the percentages of IPAQ Level of physical activity are computed by row, i.e. over the total number of patients in the low, moderate, severe classes respectively

For SGRQ, CASIS and HADS scores T-test p-values (pts with symptoms vs. pts. without symptoms) are shown. Chi-square test p-values of GOLD combined COPD assessment vs. presence/absence of (early-morning, day-time, night-time) symptoms are shown. Fisher exact test p-values of IPAQ Level of physical activity vs. presence/absence of (early-morning, day-time, night-time) symptoms are shown. P-values < 0.0005 (alpha adjusted for multiple comparisons) are in **bold**

*CASIS* COPD and Asthma Sleep Impact Scale. *CB* Chronic Bronchitis. *EM* Emphysema. *HADS* Hospital Anxiety and Depression Scale. *IPAQ* International Physical Activity Questionnaire. *SGRQ* St. George’s Respiratory Questionnaire

As Table 4 shows, independently of clinical phenotype, the presence of symptoms during the 24-h day was associated with lower QoL (T-test p-values SGRQ scores for patients with vs. without symptoms < 0.0005) and worse quality of sleep (T-test p-values CASIS score patients with vs without symptoms < 0.0005).

In both the CB and EM phenotypes, mood disturbances were significantly worse in patients with day-time and night-time symptoms: higher HADS scores were observed, in fact, in patients with vs. without symptoms (T-test p-values HADS scores patients with vs. without symptoms < 0.0005). Lastly, the level of physical activity did not differ significantly between CB or EM patients with early-morning, day-time or night-time symptoms and those without them (see Table 4).

## Discussion

The observations reported here indicate that a very high proportion of individuals suffering from COPD and under chronic inhaled medication nevertheless still show symptoms during some parts of the day. In particular, almost 80% of COPD subjects experienced symptoms during day-time or early-morning, and half had nocturnal symptoms. The novelty of this study is the relationship between circadian rhythm of symptoms and the pre-defined clinical phenotypes. Night-time symptoms, alone or in combination with symptoms during other parts of the day, appear to characterize the CB phenotype specifically.

A previous multicenter observational investigation (ASSESS study) was conducted in European clinical practice centers to determine the prevalence and severity of respiratory symptoms during different parts of the 24-h day and other patient-reported outcomes (PROs) [12]. That study found that more than half of COPD patients had symptoms during the 24-h day, which were associated with worse PROs; interestingly, two thirds of these patients experienced night-time symptoms. The present study confirms and extends these observations: in a similar cohort of COPD subjects, nocturnal symptoms were confirmed to be very common and more frequent in the CB than in the EM phenotype. This finding advances our knowledge on the distinctive clinical appearance of COPD patients. Whether this is of clinical importance has yet to be demonstrated, and future confirmatory studies are required. In both CB and EM groups, night-time symptoms were associated with worse quality of life and quality of sleep and higher levels of anxiety/depression.

From a clinical standpoint, there is an urgent need to move away from the simplistic definition of COPD embracing the entire spectrum of individuals with chronic, non-reversible airway obstruction and to develop instead a classification of subgroups on the basis of common pathogenetic mechanisms and similar clinical manifestations. The commonly accepted concept of COPD is well described by the Venn diagram, which covers different clinical phenotypes and their overlap conditions [25], in the effort to classify COPD patients into distinct clinical manifestations, each of which requires specific treatment. In the present study, researchers were asked for a clinical diagnosis of the phenotype of consecutively enrolled COPD individuals based on their judgment (according to medical history and clinical characteristics). Chest imaging and lung function, when available, helped to determine or confirm the clinical decision. In this respect, the GOLD document [11] focuses mainly on symptoms and future risk in order to determine the proper treatment, with no distinction into clinical phenotypes. This missing information is likely have been a factor in the failure of large interventional clinical trials. There is no doubt that COPD patients may respond selectively to different treatments, depending on their functional and clinical manifestations, and the recent literature offers evidence in support of this concept [26–29]. The identification of clinical phenotypes, therefore, may entail prognostic consequences at the individual patient level. Since current treatment is not sufficient to control nocturnal symptoms, specific actions are strongly advocated, either a more aggressive pharmacological approach or tests of the efficacy of non-pharmacological interventions, such as pulmonary rehabilitation or non-invasive ventilation; the latter have never been investigated and warrant specially designed ad hoc studies.

The reasons for the greater prevalence of nighttime symptoms in CB individuals are unclear. Bronchial hypersecretion and the consequent accumulation of mucus during the night probably explains, at least in part, the more frequent occurrence of nocturnal symptoms. Also, the occurrence of expiratory flow limitation (EFL) at night could contribute to the respiratory symptoms. Indeed, increased bronchial vagal hypertone, typically occurring at night, and chronic inflammation and mucus, together with changes in lung volume owing to the supine position, can favor EFL, which in turn causes air trapping and pulmonary hyperinflation. In addition, cough – which is the cardinal symptom of CB – is a common cause of nocturnal awakening. On the other hand, dyspnea is typically effort-related in mild to moderate EM and is accordingly less severe, when the patient is at rest. Finally, one cannot rule out report bias on the part of EM patients. Indeed, night-time respiratory distress, by increasing the abdominal pressure, might induce nicturia, and this (rather than dyspnea) might, in turn, be perceived as the cause of night-time awakening, along the lines of what has been observed in elderly OSAS patients [30].

The lack of differences between the clinical phenotypes examined with specific regard to quality of life, mood changes and physical performance is somewhat surprising, and it implies clinical consequences. However, the similarities in terms of lung function impairment and dyspnea perception may have played at least some role in explaining the observed findings. It cannot be excluded that missing data for each variable explored affected the outcomes. Our present findings should improve awareness of night-time symptoms in COPD individuals. Thus, in clinical practice the occurrence of symptoms such as wheezing and coughing during the night should be subjected to routine investigation. The automated systems of long-term monitoring of respiratory symptoms now available can record respiratory symptoms during the nocturnal hours that would otherwise go unnoticed [31].

Interestingly, the present study found no significant difference in level of physical activity, in either the CB or the EM group, between subjects with and without early-morning, day-time or night-time symptoms. This conflicts with previous studies according to which morning symptoms are responsible for limitations of physical activity and a consequent sedentary lifestyle [32, 33]. Differences between the tools used to assess morning symptoms and those used to assess the level of physical activity, as well as the items derived by the latter to perform the analysis, may help to explain the discordance between this study and those others.

The main limitation of the present study is that the STORICO study was not specifically designed to investigate differences between clinical phenotypes, so no hypotheses on the proper sizes of the groups were made a priori. As a consequence, only the CB and EM phenotypes had a large enough sample and no conclusions could be drawn with regard to MCA. Another limitation is the lack of available spirometries in a minority of the subjects enrolled. The careful evaluation of the medical history and previous functional assessment of these subjects enhanced the probability of diagnosis of COPD. Furthermore, since lung function assessment was not included among the primary outcomes of the protocol, the main findings of the study were not affected by it. Recall bias could also have affected data collection: this is why the *Night-time, Morning and Day-time Symptoms of COPD questionnaire,* with its short recall period of only 1 week, was chosen to evaluate the primary objectives. Other information was usually available in medical charts or could be drawn from routine questionnaires filled out during visits.

## Conclusions

In conclusion, the findings of this study indicate that the circadian rhythm of symptoms can supplement the classical phenotyping of COPD patients by revealing the prevalent phenotype-specific patterns of symptoms. Pending a confirmatory study, these findings seem robust enough to recommend systematic assessment of nocturnal symptoms and their correlates, mainly anxiety and depression, in the clinical assessment of COPD patients. This would improve our knowledge of patients’ health status and, hopefully, would help to tailor COPD therapy to the individual patient.

## Data Availability

The data that support the findings of this study are available from authors and Laboratori Guidotti and Malesci but restrictions apply to the availability of these data, which were used under license for the current study, and so are not publicly available. Data are however available from the authors and Laboratori Guidotti upon reasonable request. Data request may be sent to the first author (nicola.scichilone@unipa.it) and to Stefania Barsanti (Laboratori Guidotti, sbarsanti@labguidotti.it).

## Abbreviations

ACO: Asthma-COPD overlap
ATS: American Thoracic Society
BMI: Body mass index
CASIS: COPD and Asthma Sleep Impact Scale
CB: Chronic bronchitis
COPD: Chronic Obstructive Pulmonary Disease
DLCO: Diffusion capacity for carbon monoxide
EFL: Expiratory flow limitation
eGFR: estimated glomerular filtration rate
EM: Emphysema
ERS: European Respiratory Society
FEV_1_: Forced expiratory volume in the first second
FVC: Forced vital capacity
GERD: Gastroesophageal Reflux Disease
GOLD: Global Initiative for Chronic Obstructive Lung Disease
HADS: Hospital Anxiety and Depression Scale
HR-QoL: Health-related quality of life
IC: Inspiratory capacity
ICS: Inhaled corticosteroids
IPAQ: International Physical Activity Questionnaire
IQR: Interquartile range
LABA: Long-acting beta-agonist
LAMA: Long-acting muscarinic antagonists
MCA: Mixed COPD-asthma
mMRC: Modified Medical Research Council
NK: Unknown
PROs: Patient reported outcomes
RV: Residual Volume
SD: Standard deviation
SGRQ: St. George’s Respiratory Questionnaire
TLC: Total Lung Capacity
