# Correction to: Circadian rhythm of COPD symptoms in clinically based phenotypes. Results from the STORICO Italian observational study

**DOI:** 10.1186/s12890-019-0992-6

**Published:** 2019-12-04

**Authors:** Nicola Scichilone, Raffaele Antonelli Incalzi, Francesco Blasi, Pietro Schino, Giuseppina Cuttitta, Alessandro Zullo, Alessandra Ori, Giorgio Walter Canonica, on behalf of STORICO study group

**Affiliations:** 1 DIBIMIS, University of Palermo, Piazza delle Cliniche, 2, 90127 Palermo, Italy; 2 University Biomedical Campus of Rome, via Alvaro del Portillo, 21, 00128 Rome, Italy; 3 Internal Medicine Department, Respiratory Unit and Cystic Fibrosis Adult Center Fondazione IRCCS Cà Granda Ospedale Maggiore Policlinico and Department of Pathophysiology and Transplantation, University of Milan, via Francesco Sforza, 35, 20122 Milan, Italy; 4 Miulli Hospital, Acquaviva delle FontiStrada Prov. 127 Acquaviva – Santeramo Km. 4, 10070021 Bari, Italy; 5 National Research Council, via Ugo La Malfa, 153, 90146 Palermo, Italy; 6 Medineos Observational Research, Viale Virgilio 54/U, 41123 Modena, Italy; 7 Personalized Medicine Asthma and Allergy Clinic Humanitas University Humanitas research Hospital Rozzano (Milan), via Manzoni, 56, 20089 Rozzano, MI Italy

**Correction to: Pulm Med (2019) 19:171**

**https://doi.org/10.1186/s12890-019-0935-2**

Following publication of the original article [1], the authors flagged that the article had been provided with the names of the authors (not including the STORICO study group) in the wrong order: the ‘Given Names’ and Family Names’ were erroneously swapped around.

Please see this table for the correct order of the names:

**Table:** 

Family Name	Given name
Scichilone	Nicola
Antonelli Incalzi	Raffaele
Blasi	Francesco
Schino	Pietro
Cuttitta	Giuseppina
Zullo	Alessandro
Ori	Alessandra
Canonica	Giorgio Walter

This error has now been corrected in the original article and the corrected author list is detailed in this article.
